# *Combretum quadrangulare* Extract Attenuates Atopic Dermatitis-Like Skin Lesions through Modulation of MAPK Signaling in BALB/c Mice

**DOI:** 10.3390/molecules25082003

**Published:** 2020-04-24

**Authors:** Ju-Hyoung Park, Min Hee Hwang, Young-Rak Cho, Seong Su Hong, Jae-Shin Kang, Won Hee Kim, Seung Hwan Yang, Dong-Wan Seo, Joa Sub Oh, Eun-Kyung Ahn

**Affiliations:** 1 Department of Pharmacy, College of Pharmacy, Dankook University, Dandae-ro 119, Dongnam, Cheonan, Chungnam 31116, Korea; 2 Bio-Center, Gyeonggido Business and Science Accelerator, Gwanggyo-ro 147, Yeoungtong, Suwon, Gyeonggi 16229, Korea; 3 Biological Genetic Resources Utilization Division, National Institute of Biological Resources (NIBR), 42 Hwangyeong-ro, Seo-gu, Incheon 404-708, Korea; 4 Department of Biotechnology, Chonnam National University, Yeosu, Chonnam 59626, Korea

**Keywords:** *Combretum quadrangulare*, atopic dermatitis, inflammation, skin lesions, mitogen-activated protein kinase

## Abstract

Atopic dermatitis (AD) is a chronic inflammatory disease. *Combretum quadrangulare* (*C. quadrangulare*) is used as a traditional medicine to improve various pathologies in Southeast Asia. In this study, we investigated the effects of *C. quadrangulare* ethanol extract (CQ) on 1-chloro-2,4-dinitrobenzene (DNCB)-induced AD like skin lesions in BALB/c mice. After administration with CQ (100, 200, and 400 mg/kg) for 6 weeks, AD symptoms, protein expression, immunoglobulin E (IgE), thymus and activation-regulated chemokine (TARC), and ceramidase level were measured in skin lesions of DNCB-induced BALB/c mice. CQ group improved the dermatitis score, skin pH, transepidermal water loss (TEWL), and skin hydration. Furthermore, histological analysis revealed that CQ attenuated the increased epidermal thickness and infiltration of mast cells caused by DNCB. CQ also increased the expression of filaggrin, and reduced the expression of ceramidase, serum IgE level, and the number of eosinophils. CQ effectively inhibited cytokines and chemokines such as interleukin (IL)-6, IL-13, TARC, and thymic stromal lymphopoietin (TSLP) at the mRNA levels, as well as the activation of mitogen-activated protein kinase (MAPK), including extracellular signal-regulated kinase (ERK), c-jun N-terminal kinase (JNK), and p38 in the skin lesions. Taken together, these findings demonstrate that CQ may be an effective treatment of AD-like skin lesions by inhibiting the expression of inflammatory mediators via the MAPK signaling pathways.

## 1. Introduction

*Combretum*, the type genus of the family Combretaceae, comprises about 370 species of trees and shrubs and distributed in Africa and Asia [1,2]. One of them is *Combretum quadrangulare* (*C. quadrangulare*). *C. quadrangulare* has been used as an herbal medicine antipyretic, antidysenteric, and anthelmintic agent in Vietnam, Cambodia, Laos, Myanmar, and Thailand. Moreover, it is known to have antibacterial activity, cytotoxic, and anti-HIV activity [1,3,4]. Also, the plant and compounds isolated from *C. quadrangulare* extract were reported hepatoprotective activity in primary cultures mouse hepatocytes and D-GalN/LPS induced mouse model experiments [5,6]. However, there is no research on the ability of *C. quadrangulare* extract to suppress atopic dermatitis (AD)-like disease in vitro and in vivo.

AD is an inflammatory skin disease with itching that is characterized by repeated cycles of recovery and deterioration without specific reasons. Primary physical symptoms of AD include xerosis, lichenification, and eczematous lesions [7,8,9]. AD is generally associated with elevated immunoglobulin E (IgE), which is also related to atopic march. This refers to the sequential progress of clinical signs of atopic disease as it grows [10,11]. The clinical signs of AD in atopic march are generally known as predecessors of subsequent allergic diseases such as food allergy, asthma, and allergic rhinitis [9,12]. There are currently a few treatments for AD. Basic methods to protect damaged skin, and supplementary treatments such as hydration, are essential. New treatment methods for dermatitis, and the identification and removal of inflammatory agents, are needed. The purposes of treating AD are to maintain a healthy skin barrier and to normalize the immune and inflammatory responses of the skin [13,14]. A healthy skin barrier not only reduces water loss through the skin, but also prevents invasion of various stimuli, antigens, and infectious agents on the skin [15].

Mast cells play a very important role in inflammatory diseases and the release inflammatory mediators such as histamine, cytokines, and chemokines. In allergic skin reactions, IgE activates mast cells to induce inflammatory cytokines and the inflammatory response. Early in the progression of AD, extrinsic or environmental factors damage the skin barrier, and this damage induces serious forms of inflammatory skin disease and immune responses by IgE-mediated sensitization. AD also induces an immune system imbalance between T-helper (Th)-1 and Th2 cells. AD is commonly associated with increased serum IgE level, inflammatory chemokines, and cytokines including interleukin (IL)-4 and IL-5 [16,17,18,19]. During the progression of chronic AD, these chemokines and cytokines aggravate the inflammatory response in AD skin lesions and recruit inflammatory cells associated with the development of AD. The expression of inflammatory mediators, such as thymic stromal lymphopoietin (TSLP) and mitogen-activated protein kinase (MAPK), is associated with AD inflammatory disease. In addition, epidermal thickness and the infiltration of mast cells are increased in damaged skin lesions [20,21,22]. In this study, we investigated for the first time the effect of *Combretum quadrangulare* ethanol extract (CQ) on AD-like skin lesions in 1-chloro-2,4-dinitrobenzene (DNCB)-induced BALB/c mice.

## 2. Results

### 2.1. Effect of CQ on DNCB-Induced Ikin Lesions

**Figure 1:**
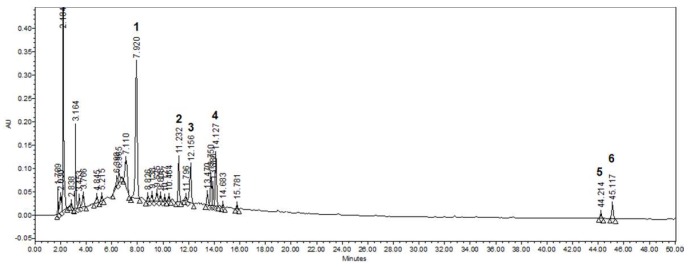
HPLC-DAD chromatogram (254 nm) of *Combretum quadrangulare* ethanol extract (CQ). Compounds are identified in the figure by number: 1 (tR = 7.92 min)—casuarinin, 2 (tR = 11.23 min)—isoorientin A, 3 (tR = 12.16 min)—orientin, 4 (tR = 14.13 min)—ellagic acid, 5 (tR = 44.21 min)—kamatakenin, 6 (tR = 45.12 min)—ayanin.

**Figure 2:**
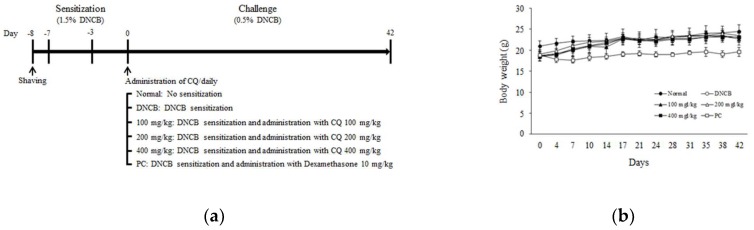
Experimental schematic diagram and change of the body weight. (**a**) After 1 week acclimation, the dorsal part to induce AD-like skin lesions was removed. The shaved dorsal skin was sensitized with 200 μL application of 1.5% 1-chloro-2,4-dinitrobenzene (DNCB) dissolved in mixture of acetone/corn oil (3:1). Thereafter, 150 μL application of 0.5% DNCB was challenged 3 times a week for 6 weeks. The mice were randomly divided into six groups with 10 mice per group (5 mice per cage): normal, DNCB, DNCB + CQ (100, 200, and 400 mg/kg) and DNCB + PC 10 mg/kg. CQ was orally administered for 6 weeks; (**b**) Mice of all groups were provided food and water ad libitum for the experiment. The body weight change was recorded twice a week.

The dried leaves and stems of *C. quadrangulare* KURZ were extracted with 50% EtOH and then analyzed using HPLC-DAD chromatogram (254 nm). As presented in Figure 1, CQ contained various compounds such as casuarinin, isoorientin A, orientin, ellagic acid, kamatakeni, and ayanin. The shaved dorsal skin was applied with DNCB to induce AD-like skin lesions for 6 weeks. The mice were administered distilled water or CQ (100, 200, and 400 mg/kg) or dexamethasone (10 mg/kg), which is used to treat variety inflammatory reactions such as, allergic disorder and AD-like diseases [23], orally for 6 weeks (Figure 2a). CQ had no effect on body weight compared to the normal group. Moreover, it was confirmed that CQ was not toxic through non animal death (Figure 2b).

**Figure 3:**
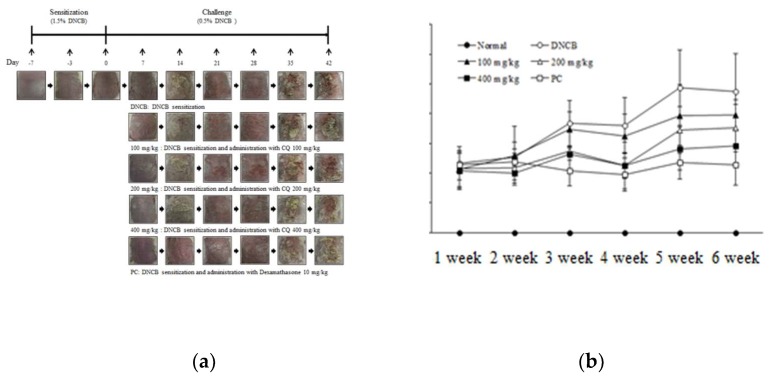
The severity on dorsal skin lesions in DNCB-induced BALB/c mice. (**a**) To demonstrate the effect of CQ in DNCB-induced BALB/c mice, clinical assessment of the dorsal skin lesions was performed once a week. The mice were randomly divided into six groups: normal, DNCB, and DNCB + CQ (100, 200, and 400 mg/kg) and DNCB + PC 10 mg/kg for 6 weeks; (**b**) The dermatitis score including erythema, dryness, edema, and erosion was calculated once a week.

To determine the dermatitis severity, pictures of the dorsal skin lesions were taken and evaluated. Dermatitis score was calculated to assess the severity of DNCB-induced AD- skin lesions in BALB/c mice (Figure 3a). The dermatitis score of the DNCB-induced group was increased compared with the other group. Importantly, CQ treatment improved the AD-like skin lesions, and the dermatitis score was attenuated compared with the DNCB-induced group (Figure 3b). Skin barrier function parameters including pH, hydration, and transepidermal water loss (TEWL) were also measured. Skin pH and TEWL were increased in the DNCB compared with the normal group, and CQ treatment decreased skin pH and TEWL levels compared with the DNCB group (Table 1 and Table 2). Skin hydration was reduced in the DNCB group compared with the normal group. CQ treatment improved the skin hydration level compared with the DNCB group (Table 3).

**Figure 4:**
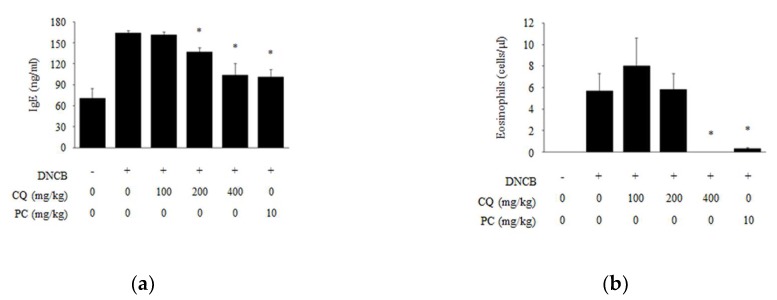
The expression of immunoglobulin E (IgE) and eosinophils in DNCB-induced BALB/c mice. (**a**) To demonstrate the effect of CQ in DNCB-induced BALB/c mice, blood was collected and serum was separated at the end of the experiment. The serum IgE level was analyzed with ELISA; (**b**) The number of eosinophil in whole blood was counted randomly. The mice were randomly divided into six groups: normal, DNCB, and DNCB + CQ (100, 200, and 400 mg/kg) and DNCB + PC 10 mg/kg for 6 weeks. All data are presented as mean ± SD; ^*^ p < 0.05 vs. DNCB-induced group.

Increased serum IgE and the presence of eosinophils are typical characteristics in AD-like disease. We observed the effects of CQ on IgE level and the number of eosinophils. Serum IgE level and the number of eosinophils were increased in the DNCB compared to normal group, whereas CQ treatment significantly reduced both serum IgE level and eosinophil numbers (Figure 4). As shown in Figure 2, Figure 3, Figure 4, the CQ treatment group (400 mg/kg) showed similar results with dexamethasone treated group as the positive control (PC).

### 2.2. Effects of CQ on Histopathological Change and Skin Barrier Dysfunction

**Figure 5:**
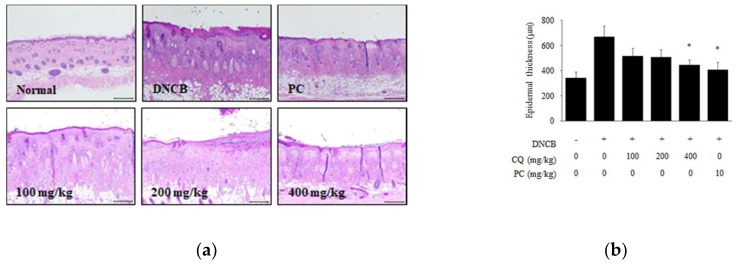

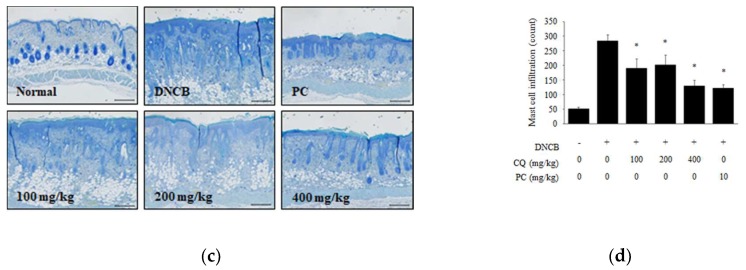
Histological analysis of epidermal thickness and mast cell infiltration on DNCB-induced skin lesions. (**a**,**b**) To observe epidermal thickness, dorsal skin section was stained with H&E and taken using a light microscope at ×100 magnification (scale bar = 100 μm); (**c**,**d**) To observe mast cell infiltration, dorsal skin section was stained with TB and taken using a light microscope at ×100 magnification (scale bar = 100 μm). All data are presented as mean ± SD; ^*^ p < 0.05 vs. DNCB-induced group.

To determine whether CQ affected histological change of DNCB-induced skin lesions, we stained dorsal skin with hematoxylin and eosin (H&E). The epidermal thickness was increased in the DNCB-induced group compared with normal group. Treatment with 400 mg/kg of CQ significantly reduced the epidermal thickness (Figure 5a,b). In addition, we stained dorsal skin with toluidine blue (TB) to observe the extent of mast cell infiltration. Mast cell infiltration in skin lesions was increased in the DNCB-induced group, but CQ treatment markedly decreased this infiltration (Figure 5c,d). Additionally, the CQ treatment group (400 mg/kg) showed similar results with dexamethasone treated group as the PC (Figure 5).

**Figure 6:**
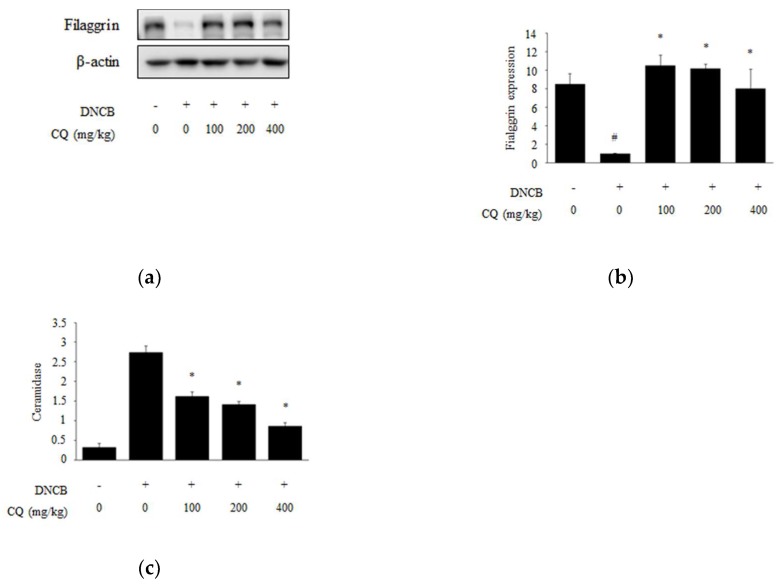
The expression of filaggrin and ceramidase on dorsal skin lesions in DNCB-induced BALB/c mice. (**a**) To demonstrate the effect of CQ in DNCB-induced BALB/c mice, the tissue was collected, homogenized, and lysed in lysis buffer at the end of the experiment. The expression of filaggrin was analyzed by Western blotting; (**b**) The relative expression level of filaggrin was measured using an image analyzer; (**c**) The level of ceramidase was analyzed with ELISA. The mice were randomly divided into five groups: normal, DNCB, and DNCB + CQ (100, 200, and 400 mg/kg) for 6 weeks. All data are presented as mean ± SD; *^#^ p <* 0.05 vs. normal group, ^*^ p < 0.05 vs. DNCB-induced group.

Filaggrin plays an important role in maintaining moisture and the skin barrier function, while ceramide, a lipid degraded by ceramidase, performs similar functions. When deficiencies of filaggrin and ceramide impair skin barrier functions, the skin becomes rough and loses transparency, resulting in dry skin [24,25,26]. The expression of filaggrin in DNCB-induced skin lesions was significantly decreased compared with the normal group, but all concentration of CQ (100, 200, and 400 mg/kg) increased its expression (Figure 6a,b). Ceramidase was sharply increased in the DNCB-induced group compared with the normal group, but CQ treatment dose-dependently decreased the expression of this enzyme (Figure 6c).

### 2.3. Effects of CQ on Release of Proinflammatory Cytokines and Chemokines

**Figure 7:**
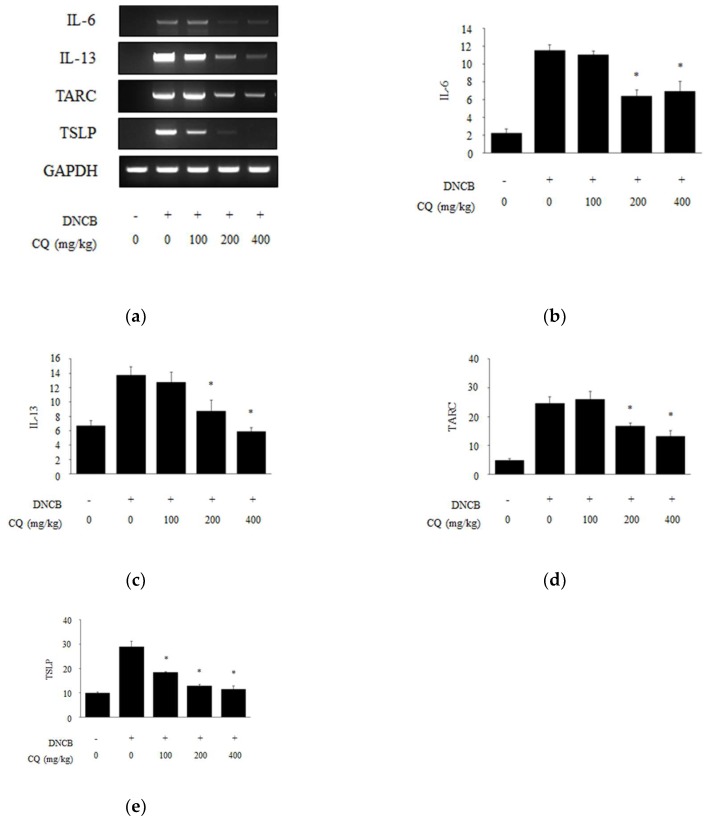
Proinflammatory cytokines and chemokines in DNCB-induced BALB/c mice. (**a**) To demonstrate the effect of CQ in DNCB-induced BALB/c mice, the tissue was collected and homogenized and the total RNA was extracted at the end of the experiment; (**b–e**) The expression of IL-6, IL-13, thymus and activation-regulated chemokine (TARC), and thymic stromal lymphopoietin (TSLP) was measured by reverse transcription polymerase chain reaction (RT-PCR) and densitometry protocol. All data are presented as mean ± SD; ^*^ p < 0.05 vs. DNCB-induced group.

Proinflammatory cytokines and chemokines including IL-6, IL-13, and thymus and activation-regulated chemokine (TARC), are released in inflammatory diseases [27]. To determine the effects of CQ on an AD-like disease, these inflammatory cytokines and chemokines were measured in DNCB-induced skin lesions at the mRNA level. The expression of IL-6, IL-13, and TARC mRNAs was increased in the DNCB-induced group compared with the normal group. Treatment with 200 and 400 mg/kg of CQ significantly suppressed the mRNA levels of all three factors in DNCB-induced skin lesions (Figure 7a–d). We also measured the expression of TSLP at the mRNA level and showed. CQ treatment effectively suppressed its expression in a dose-dependent (Figure 7a,e).

### 2.4. Effect of CQ on the Phosphorylation of MAPK Pathways on DNCB-Induced Skin Lesions

**Figure 8:**
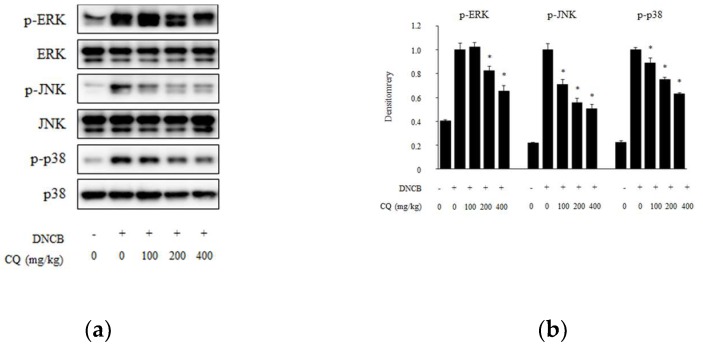
Phosphorylation of mitogen-activated protein kinase (MAPK) on dorsal skin lesions in DNCB-induced BALB/c mice. (**a**) To demonstrate the effect of CQ in DNCB-induced BALB/c mice, the tissues were collected, homogenized, and lysed in ice-cold lysis buffer at the end of the experiment. The expression of total protein was measured by western blotting; (**b**) The activities of phospho (p)-ERK (extracellular signal-regulated kinase), p-JNK (c-jun N-terminal kinase), and p-p38 were determined by densitometry protocol. All data are presented as mean ± SD; ^*^ p < 0.05 vs. DNCB-induced group.

Phosphorylation of MAPK induces an inflammatory response. Thus, we observed whether CQ regulated MAPK signaling pathways, specifically extracellular signal-regulated kinase (ERK), c-jun N-terminal kinase (JNK), and p38. As shown in Figure 8, the phosphorylation of these MAPK was increased in DNCB-induced skin lesions compared with the normal group. CQ treatment dose-dependently inhibited DNCB-induced phospholation of MAPK. The activation of ERK was remarkably reduced by treatment with 200 and 400 mg/kg of CQ, whereas the activation of JNK and p38 was significantly reduced at all concentration of CQ (100, 200, and 400 mg/kg).

## 3. Discussion

**Figure A1:**
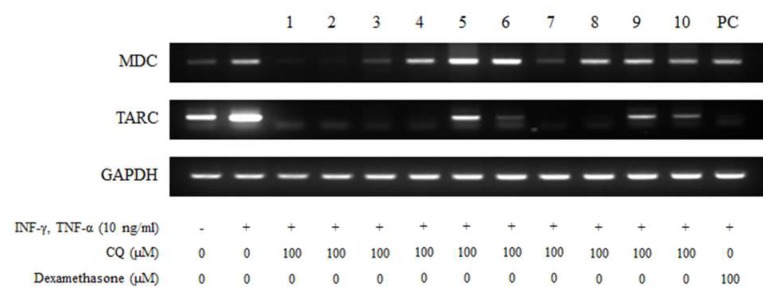
Effect of CQ on the expression of chemokines in HaCaT cells. HaCaT cells were treated with 100 μM CQ and stimulated with IFN-γ and TNF-α (10 ng/mL) for 24 h. The mRNA expression of MDC and TARC was analyzed using RT-PCR in HaCaT cells. Dexamethasone was used as a positive control (PC). 1. Ayanin, 2. 3,7-Dimethoxy-5,3′,4′-trihydroxyflavone, 3. 3,4′-Dimethoxy-5,7,3′-trihydroxyflavone, 4. Kamatakenin, 5. Unknown, 6. Kamatakenin, 7. Unknown, 8. Retusin, 9. Casuarinin, 10. Casuarinin.

The leaves, stem, and seeds of *C. quadrangulare* have long been used as traditional medicine in Southeast Asia. Previous studies on *C. quadrangulare* have mainly reported the hepatoprotective activities of its components, such as triterpenoids, sterols, and flavonoids [5,6,28]. In this study, we identified compounds consisted of CQ and analyzed various substances contained in the CQ using HPLC. As shown in Figure 1, we confirmed the presence of a variety of flavonoids and triterpenoids in the CQ. In addition, we showed that these compounds had an inhibitory effect on chemokines expression in HaCaT cells (Appendix A Figure A1). There are few studies that have explored about effects or activities of *C. quadrangulare* on AD. Therefore, this study investigated the effect of CQ on AD, which is associated with environmental risk factors and has an increasing prevalence worldwide.

AD is a chronic relapsing eczema skin disease accompanied by severe pruritus. Although the exact etiology and pathogenesis are unknown, abnormalities of epidermal penetration and antimicrobial barrier function, along with genetic and immunological factors, have been suggested as important causes of AD [29,30]. In AD, abnormalities of skin barrier functions, such as increased TEWL and skin pH, and a reduction of skin hydration, are observed. This results in AD skin having a reduced water content in the stratum corneum compared to normal skin, and water binding capacity is also reduced. TEWL represents the amount of water evaporated from the skin surface, which is high in AD. A weakly acidic pH is very important to maintain the skin barrier. Deterioration of skin barrier function is associated with skin alkalinization, and skin pH tends to be higher than normal in AD [13,31,32]. TEWL and skin pH were increased, while skin hydration was decreased in DNCB-induced skin lesions. CQ treatment alleviated these abnormalities of skin barrier function by facilitating changes in TEWL, skin pH, and skin hydration (Table 1, Table 2 and Table 3).

Mast cells that secrete inflammatory mediator cytokines play a major role in inflammation. IgE binds to the surface of mast cells with high-affinity for IgE-Fc receptor type I, resulting in the release of various types of inflammatory mediators such as chemokines and cytokines [30,33,34,35]. As shown in Figure 4 and Figure 5, CQ treatment reduced the level of serum IgE and the number of eosinophils, as well as mast cells infiltration in DNCB-induced skin lesions compared to those in DNCB alone controls.

Filaggrin is a major structural protein in the stratum corneum. Various proteases break down filaggrin, and the degradation products are moisturizing factors that play important roles in maintaining skin moisture, skin pH, and permeability of the skin barrier. Ceramide, the main component that forms lipid membranes between keratinocytes, is a water-retaining molecule that prevents water loss and pathogen infection. Ceramidase is an enzyme that cleaves ceramide [36,37,38,39,40]. We observed that CQ treatment increased filaggrin expression and reduced ceramidase expression, leading to some repair of skin barrier functions (Figure 6).

In the pathogenesis of AD, Th2 immune responses play important roles, and Th2 cytokines via Th2 immune responses cause allergic reactions [33,34,35]. The expression of IL-4, IL-6, and IL-13, which are a type of Th2 cytokines, is related to inflammatory reactions. Previous reports have shown that Korean red ginseng water extract, *Centella asiatica* extract, and esculetin from *Fraxinus rhynchophylla* have an inhibitory effect on Th2 cytokines expression in the AD-like lesion or HaCaT cells [41,42,43]. We also showed that CQ treatment inhibited the expression of Th2 cytokines, such as IL-6 and IL-13, in DNCB-induced skin lesions (Figure 7b,c). TSLP, an important factor in the pathogenesis of AD, affects inflammatory cells such as mast cells, basophils, and eosinophils, and activates dendritic cells to induce Th2 immune responses. TARC secreted by dendritic cells is a chemokine that plays a role in attracting Th2 cells to the site of inflammation in skin lesions [44,45]. The expression of TARC and TSLP were increased in AD-like skin lesions and, importantly, CQ treatment reduced the expression of both factors (Figure 7d,e).

MAPK signaling pathways are involved in intracellular inflammatory responses. Through the inflammatory response of various immune cells, the activation of MAPK, including ERK, JNK, and p38, increases proinflammatory cytokines and responses in the intracellular pathway [46,47]. Our previous study reported that Korea red ginseng extract improves AD-like inflammatory response via inhibition of ERK 1/2, JNK, and p38 phosphorylation [41]. In this study, the phosphorylation of MAPK was increased in AD-like skin lesions compared to that in the normal skin. However, CQ treatment inhibited the phosphorylation of MAPK (Figure 8).

In conclusion, this is the first report to verify the important actions associated with pharmacological roles of CQ in the inflammatory response of AD-like skin lesions. Therefore, the findings of the present study suggest that CQ may be an effective agent for the prevention and treatment of AD-related disease.

## 4. Materials and Methods

### 4.1. Preparation of Plant Material

The dried leaves and stems of *C. quadrangulare* KURZ (Combretaceae) were collected from Ninh Son, Ninh Thuan, Vietnam, in March, 2016. Dr. J. S. Kang (National Institute of Biological Resources) performed the botanical identification, and a voucher specimen (#415) was deposited at the Bio-Center, Gyeonggido Business & Science Accelerator, Suwon, Republic of Korea. The dried leaves and stems of *C. quadrangulare* (1 kg) were pulverized and extracted with 50% ethanol (2 × 10 L) at room temperature for 2 days. The extract was filtered and concentrated in vacuum under reduced pressure using rotary flash evaporator (Büchi, Flawil, Switzerland) and allowed for complete evaporation of the ethanol. The remaining aqueous solution concentrated under vacuum and freeze dried (ilShinbiobase, Yangju, Korea). The yield of the crude extract was 5% (*w*/*w*).

### 4.2. HPLC Analysis

Waters Alliance e2695 separating module (Waters, MA, USA) using photodiode array detector (Waters 2998) with autosampler and column oven was used for the analysis. Separation was achieved using a Kromasil 100-5-C18 column (5 μm, 250 × 4.6 mm i.d., AkzoNobel, Bohus, Sweden). The mobile phase consisted of water–trifluoroacetic acid (TFA, 99.95:0.05; *v*/*v*) (solvent A) and acetonitrile (solvent B). The elution was performed using the following gradient: initial 90:10 (A:B *v*/*v*); 40 min 50:50 (A:B *v*/*v*); and 60 min 0:100 (A:B *v*/*v*). The mobile phase flow rate was 1.0 mL/min. The injection volume was 10 μL, and the column temperature was set at 30 °C. All operations, including data acquisition and analysis, were controlled by Empower™ 3 chromatography data software (Waters Co., MA, USA).

### 4.3. Animals

Five weeks old Male BALB/c mice (18–22 g) were purchased from Orient, Inc. (Seoul, Republic of Korea) and acclimated to standard condition with light (12 h light-dark cycle), temperature (18–22 °C) and humidity (35–55%) in specific pathogen free environment. All experimental approval procedures were established by the Institutional Animal Care and Ethical Use Committee of the Gyeonggido Business and Science Accelerator (Approval No. 2018–09-0002) in accordance with AAALAC-International guidelines.

### 4.4. Experimental Design

To induce AD-like skin lesions, mice were anesthetized with isoflurane (HanaPharm, Seoul, Republic of Korea). Then, the back hair was removed by an electric shaver with shaving cream (Oxy Reckitt Benckiser, Chartes, France). After 1 week acclimation, the shaved dorsal skin (1 × 2 cm) was sensitized with 200 μL application of 1.5% DNCB dissolved in mixture of acetone/corn oil (3:1). Thereafter, 150 μL application of 0.5% DNCB was challenged 3 times a week for 6 weeks. The mice were randomly divided into six groups with 10 mice per group (5 mice per cage): normal, DNCB, DNCB + CQ 100 mg/kg (2 mg/200 μL), DNCB + CQ 200 mg/kg (4 mg/200 μL), DNCB + CQ 400 mg/kg (8 mg/200 μL) and DNCB + dexamethasone 10 mg/kg (Merck, Darmstadt, Germany). CQ, dissolved in distilled water, was orally administered daily for 6 weeks. The DNCB group was administered only distilled water without CQ. The mice were provided food and water ad libitum, and body weights were recorded twice a week for the experiment.

### 4.5. Evaluation of Skin Lesions and Dermatitis Score

The mice were anesthetized with isoflurane and pictures of the shaved dorsal skin and the dermatitis score were evaluated once a week. The total dermatitis score based on various symptoms such as erythema, dryness, edema, and erosion was evaluated by sum of these symptoms scores from 0 (none) to 3 (severe). In addition, skin pH, skin hydration and TEWL were measured in the DNCB-induced dorsal skin lesions to evaluate the skin clinical assessment. Skin pH of the dorsal skin was measured using a pH meter (HANNA Instruments, RI, USA), and skin hydration and TEWL of the dorsal skin were measured using a skin moisture meter (Cortex Technology, Hadsund, Denmark) weekly.

### 4.6. Analysis of IgE, Eosinophils, Thymus and Activation-Regulated Chemokine (TARC), and Ceramidase

On the 42nd day, the mice were anesthetized with isoflurane and blood samples were collected. Blood was centrifuged for 15 min at 890× *g* to collect serum that was used to measure the IgE level and blood eosinophils were counted using a hematology cell counter (Nihon-Koden, Tokyo, Japan). Dorsal skin tissue was collected, homogenized, and centrifuged to measure TARC, and ceramidase. The serum IgE (ab157718; Abcam, MA, USA), TARC (MCC170; R&D Systems, MN, USA), and ceramidase (MBS7236915; Mybiosource, CA, USA) were analyzed using enzyme-linked immunosorbent assay (ELISA) quantitation kits.

### 4.7. Western Blotting

On the 42nd day, the dorsal skin was collected, homogenized, centrifuged and the contents of total protein were measured by Bradford assay. The dorsal skin lysate was separated at 100 V and transferred onto nitrocellulose membrane (Whatman, Dassel, Germany) for 85 min at 400 mA. After transfer, the membrane was blocked in 5% bovine serum albumin for 1 h and incubated with primary antibodies against filaggrin (Enzo, Farmingdale, NY, USA), p-ERK, p-JNK, p-p38 (Cell Signaling Technology, Beverly, MA, USA), and β-actin (Santa Cruz Biotechnology, CA, USA) overnight at 4 °C. The membrane was washed with Tris-buffered saline with 0.1% Tween 20 and incubated with horseradish peroxidase-conjugated secondary antibodies (Santa Cruz Biotechnology, CA, USA) for 1 h at room temperature. The protein band was enhanced by a chemiluminescence reagent and visualized using gel imaging system (Bio-Rad, Hercules, CA, USA).

### 4.8. RT-PCR

On the 42nd day, total RNA from the dorsal skin was isolated and obtained. The single stranded complementary DNA (cDNA) was synthesized using the SuperScript®III First-Strand Synthesis System (Thermo Fisher Scientific, MA, USA) and amplified using AccuPower® Pfu PCR PreMix (Bioneer, Daejeon, Republic of Korea) in a MG96G thermal cycler (LongGene Scientific Instruments, Hangzhou, China). For amplification, RT-PCR cycling conditions were as follows: 5 min at 95 °C followed by 27 cycles of 30 s at 95 °C, 40 s at between 55 and 60 °C, 1 min at 72 °C, then 10 min at 72 °C for final extension. The primer sequences for amplification are listed in Table 4.

### 4.9. Histological Analyses

On the 42nd day, the dorsal skin was collected, fixed with 10% neutral buffered formalin and embedded in paraffin. The embedded paraffin was cut into 5 μm sections, stained with H&E, and examined using a light microscope (Nikon, Tokyo, Japan) at 100× magnification to measure epidermal thickness. These sections were stained with TB to measure mast cell infiltration and the number of mast cells determined by counting three sections randomly at 100× magnification.

### 4.10. Statistical Analysis

Statistical analysis was determined using one-way analysis of variance, followed by the Dunnett′s test. All data are presented as mean ± standard deviation (SD). ^*^ p value < 0.05 was considered statistically significant and *^#^ p value <* 0.05 was considered statistically significant.

## Figures and Tables

**Table 1:** The effect of CQ on the skin pH in DNCB-induced BALB/c mice.

Group	1 Week	2 Week	3 Week	4 Week	5 Week	6 Week
Normal	5.69 ± 0.18	5.58 ± 0.28	5.46 ± 0.16	5.52 ± 0.13	5.48 ± 0.12	5.53 ± 0.17
DNCB	6.38 ± 0.21 *^#^*	6.48 ± 0.25 *^#^*	6.45 ± 0.29 *^#^*	6.39 ± 0.27 *^#^*	6.50 ± 0.20 *^#^*	6.47 ± 0.14 *^#^*
100 mg/kg	6.24 ± 0.08	6.31 ± 0.24	6.23 ± 0.25 ^*^	6.19 ± 0.22	6.24 ± 0.17 ^*^	6.23 ± 0.18 ^*^
200 mg/kg	6.21 ± 0.19 ^*^	6.26 ± 0.27	6.17 ± 0.26 ^*^	6.21 ± 0.27	6.21 ± 0.14	6.20 ± 0.11 ^*^
400 mg/kg	6.27 ± 0.13	6.23 ± 0.07 ^*^	6.16 ± 0.28 ^*^	6.16 ± 0.22 ^*^	6.17 ± 0.15 ^*^	6.18 ± 0.16 ^*^

^1^ Skin pH was measured using the pH meter in dorsal skin once a week. All data were represented as mean ± SD. *^#^ p <* 0.05 vs. normal group, ^*^ p < 0.05 vs. DNCB-induced group.

**Table 2:** The effect of CQ on the TEWL in DNCB-induced BALB/c mice.

Group	1 Week	2 Week	3 Week	4 Week	5 Week	6 Week
Normal	4.93 ± 0.80	4.18 ± 0.60	4.05 ± 0.43	3.76 ± 0.24	4.23 ± 0.58	4.68 ± 0.80
DNCB	33.40 ± 4.80 *^#^*	31.53 ± 5.87 *^#^*	37.56 ± 6.84 *^#^*	39.19 ± 4.74 *^#^*	38.14 ± 6.59 *^#^*	38.49 ± 9.53 *^#^*
100 mg/kg	28.26 ± 2.44 ^*^	32.63 ± 5.13	35.54 ± 7.67	33.26 ± 4.77^*^	34.73 ± 4.38	36.20 ± 7.94
200 mg/kg	27.68 ± 2.57	31.33 ± 6.19	29.43 ± 4.51 ^*^	30.39 ± 4.41 ^*^	31.33 ± 6.05 ^*^	30.04 ± 8.51 ^*^
400 mg/kg	26.93 ± 3.03 ^*^	30.65 ± 2.97	29.31 ± 4.75 ^*^	28.03 ± 4.22 ^*^	28.03 ± 4.49 ^*^	26.78 ± 3.43 ^*^

^1^ Transepidermal water loss (TEWL) was measured using the skin moisture meter in dorsal skin once a week. All data were represented as mean ± SD. *^#^ p <* 0.05 vs. normal group, ^*^ p < 0.05 vs. DNCB-induced group.

**Table 3:** The effect of CQ on the skin hydration in DNCB-induced BALB/c mice.

Group	1 Week	2 Week	3 Week	4 Week	5 Week	6 Week
Normal	95.50 ± 10.34	104.30 ± 10.69	99.00 ± 10.71	98.00 ± 5.56	97.00 ± 4.83	104.90 ± 11.84
DNCB	50.80 ± 11.35 *^#^*	45.60 ± 6.57 *^#^*	48.10 ± 9.83 *^#^*	46.60 ± 9.70 *^#^*	46.80 ± 5.88 *^#^*	44.10 ± 9.42 *^#^*
100 mg/kg	50.60 ± 11.40	51.40 ± 13.58	51.90 ± 13.97	54.70 ± 10.37	52.80 ± 10.66	52.70 ± 5.79
200 mg/kg	53.30 ± 15.41	52.20 ± 14.28	53.50 ± 11.14	57.50 ± 9.72 ^*^	55.20 ± 12.11 ^*^	56.80 ± 7.24 ^*^
400 mg/kg	55.20 ± 16.87	53.40 ± 15.34	55.50 ± 6.57 ^*^	56.80 ± 10.84	54.30 ± 9.51 ^*^	56.90 ± 7.92 ^*^

^1^ Skin hydration was measured using the skin moisture meter in dorsal skin once a week. All data were represented as mean ± SD. *^#^ p <* 0.05 vs. normal group, ^*^ p < 0.05 vs. DNCB-induced group.

**Table 4:** Primers for PCR amplification.

Primer	Forward Sequence	Reverse Sequence
IL-6	5′-AAC CTT CCA AAG ATG GCT GAA -3′	5′-CAG GAA CTG GAT CAG GAC TTT-3′
IL-13	5′-CAG CTC CCT GGT TCT CTC AC-3’	5′-CCA CAC TCC ATA CCA TGC TG-3′
TSLP	5′-CGA CAG CAT GGT TCT TCT CA-3′	5′-CGA TTT GCT CGA ACT TAG CC -3′
TARC	5′-ATG GCC CCA CTG AAG ATG CT-3′	5′-TGA ACA CCA ACG GTG GAG GT-3′
GAPDH	5′-GAG GTA AAC TCA GGA GAG TG-3′	5′-GTA GAC TCC ACG ACA TAC TC-3′
